# Supplementary material for: Hybrid Models and Biological Model Reduction with PyDSTool
Source: PLoS Comput Biol. 2012 Aug 9;8(8):e1002628. doi: 10.1371/journal.pcbi.1002628 (PMC3415397; doi:10.1371/journal.pcbi.1002628)
Supplement: Text S4 — Complete source code for the PyDSTool package (version 0.88.120504). Includes API documentation and help files linking to web pages. This file is identical to the current public release on Sourceforge.net. (ZIP) [file pcbi.1002628.s004.zip › PyDSTool/html/PyDSTool.Toolbox.dssrt.dssrt_assistant-class.html]

xml version="1.0" encoding="ascii"?


PyDSTool.Toolbox.dssrt.dssrt\_assistant


| Home | Trees | Indices | Help | PyDSTool |
| --- | --- | --- | --- | --- |

Package PyDSTool ::
Package Toolbox ::
Module dssrt ::
Class dssrt\_assistant

[hide private]

[frames] | no frames]

# Class dssrt\_assistant

source code

```
object --+
         |
        dssrt_assistant
```

---


|  |  |
| --- | --- |
| Instance Methods | [hide private] |

|  |  |
| --- | --- |
| \_\_init\_\_(self, kw)  x.\_\_init\_\_(...) initializes x; see x.\_\_class\_\_.\_\_doc\_\_ for signature | source code |

|  |  |
| --- | --- |
| \_\_getstate\_\_(self) | source code |

|  |  |
| --- | --- |
| \_\_setstate\_\_(self) | source code |

|  |  |
| --- | --- |
| \_setup\_taus\_psis(self) | source code |

|  |  |
| --- | --- |
| \_init\_from\_MSpec(self, mspec) | source code |

|  |  |
| --- | --- |
| \_process\_expr(self, expr)  Create two Symbolic.Fun(ction) versions of the given expression: | source code |

|  |  |
| --- | --- |
| reset(self)  Delete current state based on given trajectory | source code |

|  |  |
| --- | --- |
| ensure(self) | source code |

|  |  |
| --- | --- |
| calc\_tau(self, target, pt)  Calculate tau of target variable directly from a given single point containing only the state variables. | source code |

|  |  |
| --- | --- |
| calc\_inf(self, target, pt)  Calculate inf of target variable directly from a given single point containing only the state variables. | source code |

|  |  |
| --- | --- |
| calc\_psi(self, target, pt, focus=None)  Calculate Psi of focus variable w.r.t. | source code |

|  |  |
| --- | --- |
| calc\_taus\_infs(self, focus=False)  Calculate taus and infs from given trajectory. | source code |

|  |  |
| --- | --- |
| calc\_Is(self)  Calculate currents from given trajectory | source code |

|  |  |
| --- | --- |
| calc\_psis(self)  Calculate Psis from given trajectory | source code |

|  |  |
| --- | --- |
| make\_pointsets(self) | source code |

|  |  |
| --- | --- |
| calc\_rankings(self, influence\_type=`'``psi``'`) | source code |

|  |  |
| --- | --- |
| reduced\_Vode\_system(self, vars)  Doesn't allow fast vars to be algebraic - better solution is to use ModelTransforms | source code |

**Inherited from `object`**:
`__delattr__`,
`__getattribute__`,
`__hash__`,
`__new__`,
`__reduce__`,
`__reduce_ex__`,
`__repr__`,
`__setattr__`,
`__str__`


|  |  |
| --- | --- |
| Properties | [hide private] |

**Inherited from `object`**:
`__class__`


|  |  |
| --- | --- |
| Method Details | [hide private] |

|  |  |
| --- | --- |
| \_\_init\_\_(self, kw)  *(Constructor)* | source code |

x.\_\_init\_\_(...) initializes x; see x.\_\_class\_\_.\_\_doc\_\_ for
signature

Overrides: object.\_\_init\_\_: *(inherited documentation)*

|  |  |
| --- | --- |
| \_process\_expr(self, expr) | source code |

```
Create two Symbolic.Fun(ction) versions of the given expression:

(1) The first is intended for processing post-integration
pointsets to yield the dominant scale Psi quantities,
when the pointsets contain already-computed auxiliary variables
for taus, infs, etc.

(2) The second is intended for on-demand calculation of Psi's
when only the system's state variables are given, thus
taus and infs etc. must be computed this class. If user did not
provide the dssrt_fn functions this will be ignored and not created.

The returned pair of pairs is
 ((arg_list_signature (1), symbolic function (1)),
  (arg_list_signature (2), symbolic function (2)))
unless None was passed (in which case None is returned).
```

|  |  |
| --- | --- |
| calc\_psi(self, target, pt, focus=None) | source code |

Calculate Psi of focus variable w.r.t. the target variable directly
from a given single point containing only the state variables.

If focus is None (default) then focus\_var attribute is assumed.

|  |  |
| --- | --- |
| calc\_taus\_infs(self, focus=False) | source code |

Calculate taus and infs from given trajectory.

If focus=True (default False), results are only returned for the
variable of focus given by focus\_var attribute

  


| Home | Trees | Indices | Help | PyDSTool |
| --- | --- | --- | --- | --- |

|  |  |
| --- | --- |
| Generated by Epydoc 3.0.1 on Fri May 4 15:24:08 2012 | http://epydoc.sourceforge.net |
